# Pandemic risk stemming from the bovine H5N1 outbreak: an account of the knowns and unknowns

**DOI:** 10.1128/jvi.00052-25

**Published:** 2025-02-27

**Authors:** Anice C. Lowen, Amy L. Baker, Andrew S. Bowman, Adolfo García-Sastre, Scott E. Hensley, Seema S. Lakdawala, Louise H. Moncla, Martha I. Nelson, Andrew Pekosz, Rebecca L. Poulson, Wendy B. Puryear, Jonathan A. Runstadler, Troy C. Sutton, S. Mark Tompkins, Richard J. Webby, The Risk Assessment Pipeline working group of the NIAID Centers of Excellence for Influenza Research and Response (CEIRR) Network

**Affiliations:** 1 Department of Microbiology and Immunology, Emory University School of Medicine 12239 , Atlanta, Georgia, USA; 2 Virus and Prion Research Unit, National Animal Disease Center, Agricultural Research Service, U.S. Department of Pathobiology, Agriculture, Ames, Iowa, USA; 3 Department of Veterinary Preventive Medicine, The Ohio State University 2647 , Columbus, Ohio, USA; 4 Department of Microbiology, Icahn School of Medicine at Mount Sinai 200769 , New York, New York, USA; 5 Global Health and Emerging Pathogens Institute, Icahn School of Medicine at Mount Sinai 5925 , New York, New York, USA; 6 Department of Medicine, Division of Infectious Diseases, Icahn School of Medicine at Mount Sinai 377569 , New York, New York, USA; 7 The Tisch Cancer Institute, Icahn School of Medicine at Mount Sinai 5925 , New York, New York, USA; 8 Department of Pathology, Molecular and Cell-Based Medicine, Icahn School of Medicine at Mount Sinai 5925 , New York, New York, USA; 9 The Icahn Genomics Institute, Icahn School of Medicine at Mount Sinai 5925 , New York, New York, USA; 10 Department of Microbiology, University of Pennsylvania 332252 , Philadelphia, Pennsylvania, USA; 11 Department of Pathobiology, School of Veterinary Medicine, The University of Pennsylvania 634332 , Philadelphia, Pennsylvania, USA; 12 National Center for Biotechnology Information, National Library of Medicine, National Institutes of Health (NIH) 2511 , Bethesda, Maryland, USA; 13 W. Harry Feinstone Department of Molecular Microbiology and Immunology, Johns Hopkins University Bloomberg School of Public Health 25802 , Baltimore, Maryland, USA; 14 Department of Population Health, University of Georgia College of Veterinary Medicine 70734 , Athens, Georgia, USA; 15 Department of Infectious Disease & Global Health, Cummings School of Veterinary Medicine at Tufts University 52289 , North Grafton, Massachusetts, USA; 16 Department of Veterinary and Biomedical Science, The Huck Institutes, The Pennsylvania State University 8082 , University Park, Pennsylvania, USA; 17 Center for Vaccines and Immunology, College of Veterinary Medicine, University of Georgia 70734 , Athens, Georgia, USA; 18 Department of Infectious Diseases, College of Veterinary Medicine, University of Georgia 551782 , Athens, Georgia, USA; 19 Department of Host-Microbe Interactions, St Jude Children's Research Hospital 5417 , Memphis, Tennessee, USA; Indiana University Bloomington, Bloomington, Indiana, USA

**Keywords:** influenza A virus, H5N1, bird flu, bovine, pandemic preparedness, risk assessment

## Abstract

H5N1 subtype influenza A viruses represent a long-standing pandemic concern. Owing to their global occurrence in poultry, humans are routinely exposed to these viruses, and hundreds of human cases have been documented worldwide since 2003. The relevant viral lineages are not static, however, and have recently undergone a massive expansion of host range and geographic distribution. Within this expansion, the introduction of H5N1 viruses into dairy cattle in the United States has spawned a novel animal-human interface. In response, public health agencies have sought to evaluate the risk of an H5N1 pandemic stemming from the bovine outbreak. These assessments draw on evidence from the field and the laboratory to score a series of recognized risk factors. As such, their utility hinges on fundamental understanding of the processes that drive pandemic emergence and the availability of relevant data. Advancing this understanding and gathering data prior to and during an outbreak are primary missions of the NIAID Centers of Excellence for Influenza Research and Response (CEIRR) Network. To further these goals and highlight the need for an invigorated response across US agencies, here, we review gaps in understanding of the dairy cattle outbreak and identify constraints on efforts to close these gaps.

## INTRODUCTION

Research has furnished valuable insight into the sources of influenza pandemics. We know that new pandemic strains derive from influenza A viruses circulating in non-human host species (1). Wild aquatic birds are the primary reservoir of these viruses, but influenza A virus lineages are sustained in several other species, including domesticated swine, birds, horses, and dogs (2, 3). Animals with which humans have frequent contact present the most likely sources of zoonotic infection and therefore pandemics (4, 5). We know that most zoonoses do not lead to pandemics, however, and that efficient transmission among human hosts is a major barrier to pandemic emergence (6, 7). Usually, pandemic influenza viruses carry mixed-lineage genomes acquired through reassortment (8). This process of viral genetic exchange allows genes from seasonal influenza A viruses, that are well adapted to support viral replication and transmission in humans, to come together with genes from non-human strains that make the resultant virus novel to the human immune system (9, 10). In fact, phylogenetic analysis of the viruses that caused the 1957, 1968, and 2009 influenza pandemics demonstrated their reassortant origins (11–13).

Our current knowledge of the factors that promote the generation of pandemic influenza viruses can be leveraged to anticipate, prioritize, and prepare for future pandemics. Indeed, well-established tools exist to formalize virus risk assessments for these purposes (14, 15). These tools were developed by teams at the US CDC and at WHO and have been applied by these groups with the goal of assessing risks posed by the global expansion since 2020 of 2.3.4.4b lineage H5N1 influenza A viruses (16, 17). Recent risk assessment exercises have focused specifically on the ongoing influenza outbreak in US dairy cattle, caused by the B3.13 genotype of 2.3.4.4b.

The bovine H5N1 outbreak has created enormous potential for human infection: infected mammary glands in dairy cattle shed high titers of virus into milk, which in turn is produced in large volumes (18–20). Bovine H5N1 virus differs from previous H5N1 virus circulating in birds by the presence of mutations associated with mammalian adaptation. Dairy workers can be exposed to spilled, sprayed, and aerosolized milk throughout their workday. US milk production takes place at an industrial scale, such that some workers of a single large dairy farm may have routine contact with thousands of lactating cattle. Exposure risk is further increased through the spread of the virus to domestic cats and other mammals on dairy farms. This robust animal-human interface is occupied by H5N1 influenza viruses in hundreds of locations across at least 16 US states as of December 2024 (21). Dozens of human infections stemming from exposure to infected cattle have been documented (22). There is little doubt that this situation poses a pandemic risk and should be addressed to reduce the exposure of humans to H5N1 infection (23–25). However, the acuity of the risk is less certain.

**Fig 1:**
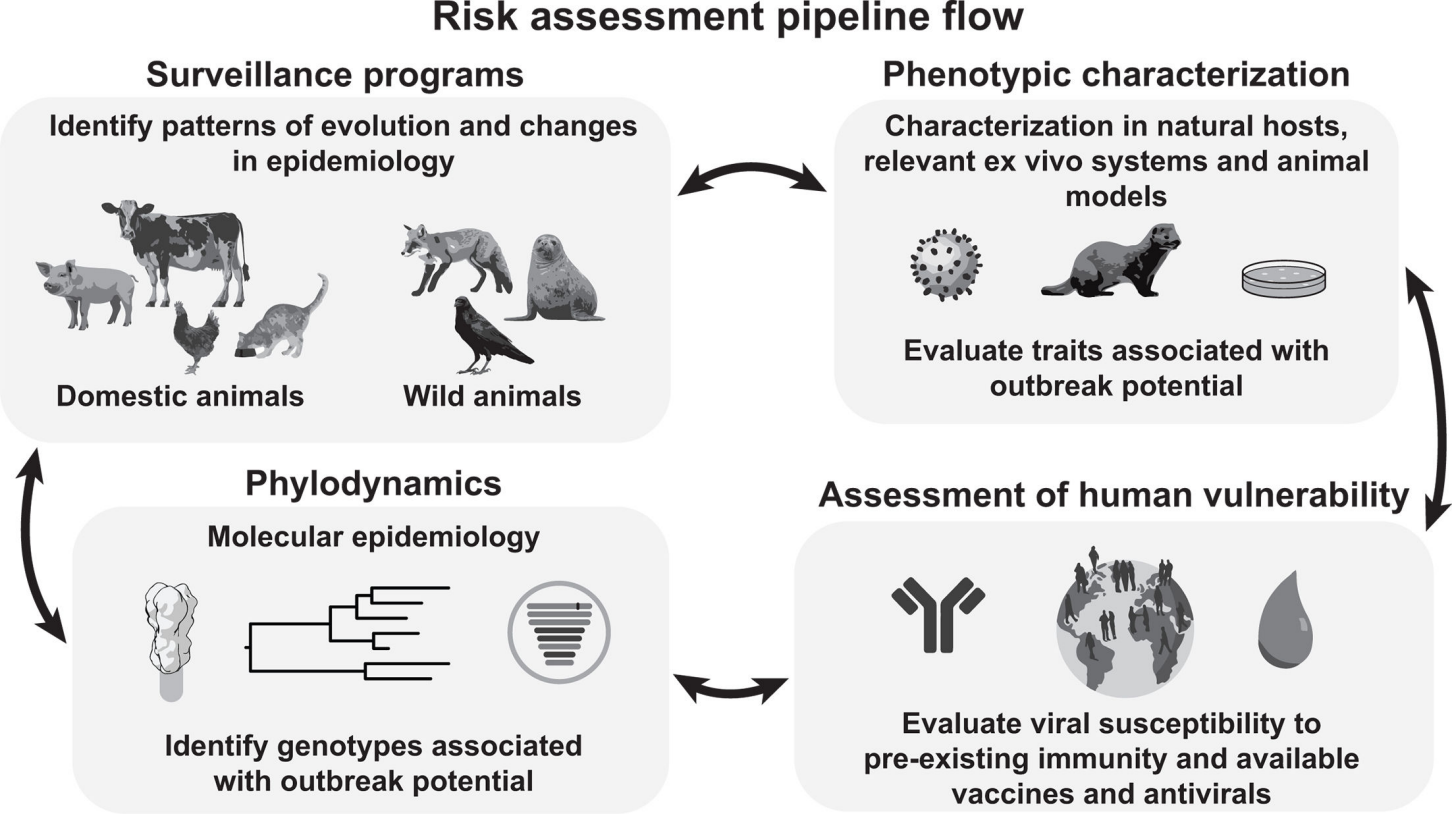
The CEIRR risk assessment pipeline seeks to advance understanding of pandemic risk through well-integrated viral surveillance, genomic characterization, phylogenetic analyses, phenotypic analyses, and evaluation of viral susceptibility to human defenses.

There is strong motivation among governments, the press, and the public to better understand the likelihood of H5N1 influenza A viruses causing a pandemic. With research funded through the Centers of Excellence for Influenza Research and Response (CEIRR) Network, we are working to support evidence-based risk assessment (Fig. 1). Here, we outline knowledge gaps that create uncertainty in assessing the pandemic risk associated with the bovine H5N1 outbreak. We then highlight limitations of current research approaches that impede efforts to fill these gaps.

## WHAT CURRENT KNOWLEDGE GAPS IMPEDE ACCURATE ASSESSMENT OF THE PANDEMIC RISK POSED BY THE BOVINE H5N1 OUTBREAK?

### Source of the dairy cattle outbreak

**Fig 2:**
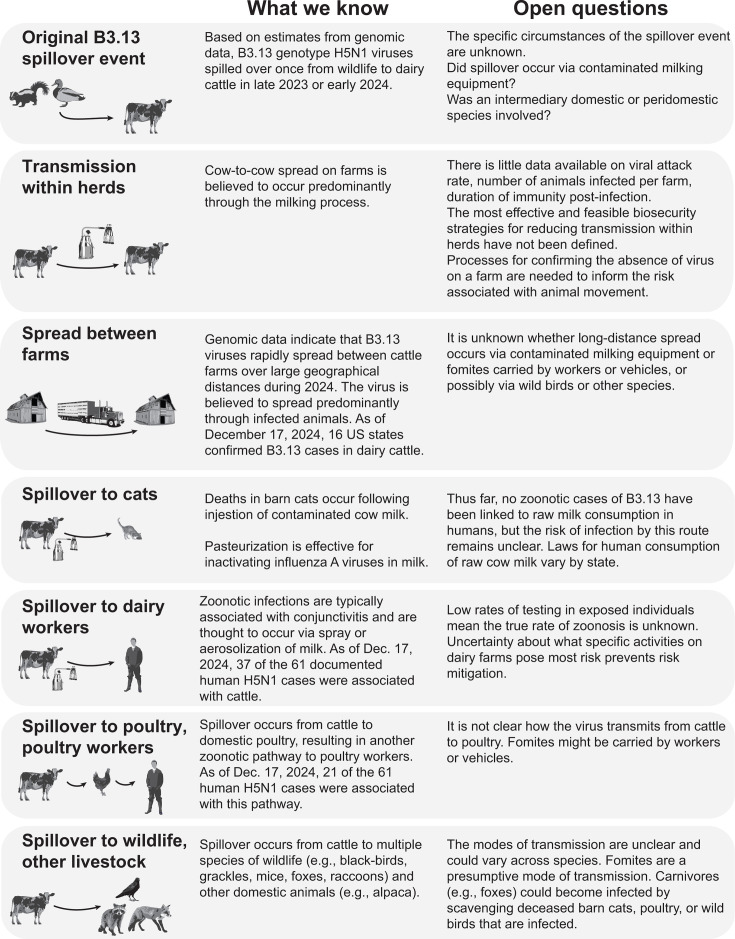
Critical open questions relate to the processes that triggered the dairy cattle outbreak present and that drive its expansion.

The H5N1 outbreak in US dairy cattle was recognized in March 2024 and is estimated to have started in late 2023 or early 2024 from a single introduction of a genotype B3.13 virus from birds to cattle (26–28). How the virus was introduced into cattle remains unknown (Fig. 2). Was there a direct introduction from wild birds? Was there an intermediate domestic or peri-domestic host? Was milking equipment the vehicle of transmission? This uncertainty is fueled by a lack of understanding of many elements of H5N1 viruses in wild birds and mammals. Owing to a paucity of surveillance, little data are available on the prevalence and genetic signatures of H5N1 viruses in wildlife near the index farms. Without this information, investigations into the mechanisms of spillover into dairy cattle are extremely limited. In turn, we are unable to assess the likelihood of similar introductions affecting other agricultural species, occurring in other countries or recurring in the USA.

In January 2025, following the initial submission of this text, a second introduction of H5N1 into US dairy cattle was detected in the state of Nevada, with multiple herds affected (29). The virus was again of the H5 2.3.4.4b lineage but was of the D1.1 genotype. While the 2.3.4.4b lineage refers to the ancestry of the hemagglutinin (HA) gene, the genotype designation describes the constellation of eight gene segments that comprise the viral genome. B3.13 and D1.1 carry different sets of gene segments derived through reassortment with low-pathogenicity avian influenza viruses. Thus, the D1.1 virus detected in Nevada was distinct from the B3.13 virus already circulating in cattle. D1.1 was prevalent in wild birds and had caused several poultry outbreaks in the weeks preceding its detection in cattle. The mode of this second introduction is again unknown, but its occurrence indicates that transfer of high-pathogenicity avian influenza to dairy cattle may be a persistent problem as long as these viruses are prevalent in the environment.

### Extent of the outbreak

A fundamental gap in our understanding of the ongoing outbreak is very simply, how big is it? Given the unprecedented nature of an influenza outbreak in cattle, early testing on farms was limited by a lack of pre-existing infrastructure. Under the Federal Order of April 2024, sample collection was mandated prior to interstate movement of dairy cattle. While this mandate has stimulated the provision of valuable samples to the National Animal Health Laboratory Network (NAHLN), the information that can be gained is limited owing to wide variation across states in sampling procedures (from no collection to systematic, active surveillance at the farm level). The Federal Order of December 2024 (30), in addition to the Dairy Herd Status Program, will improve the standardization of surveillance. At present, however, the extent of H5N1 infection in cattle is uncertain. These data gaps create significant barriers to understanding whether the outbreak is continuing to expand or is contracting, on a national scale and on more local scales, and if farms are being re-infected. In addition, the virus has been identified in rodents and cats living on dairy farms (18, 25, 31), but the frequency of infection in such peri-domestic animals and their role in driving the outbreak are poorly defined. Whether agricultural animals other than dairy cattle might be infected is also unclear, due to the lack of active surveillance systems in species other than swine and poultry. While pathogenic infections in farm animals are likely to be identified even without active surveillance, subclinical transmission can go undetected. Influenza A viruses are not conventionally known to circulate in ruminants, but the present outbreak has revealed the mammary gland as a highly permissive site of viral replication. This new understanding of influenza A virus tropism, together with the present situation in dairy cattle and H5N1 detections in Minnesota goats in early 2024 (32), suggests that routine monitoring of ruminants would be prudent, particularly if these animals comingle with susceptible avian species.

### Modes of viral dissemination driving the outbreak

As of December 2024, the B3.13 genotype had been reported in dairy cattle in 16 US states (21). While movement of cattle between farms, often across state lines, is thought to have driven much of this dispersal, questions remain. Detailed information on how cattle move across the USA is needed to understand whether animal transport is likely to be the sole driver of large-scale spread.

How the virus spreads between dairy farms and from dairy farms to poultry holdings is another important area of uncertainty. Contaminated milk discarded in the environment could play a role. Farm equipment, vehicles, and personnel may transfer the virus between locations. Infected farm workers, veterinarians, or peri-domestic animals may transmit infection. Understanding the contributions of these potential modes of spread is critical to the design of effective farm-level biosecurity that could limit the outbreak.

Viral positivity is typically reported at a farm level, without data on the number of cattle affected nor how this number changes over time and across cohorts of cattle with differing exposures. As a result, investigation of epidemiology within farms is difficult to pursue. We are left with critical gaps in understanding how the virus transmits among cattle on the same farm, with what efficiency it spreads, the frequency with which affected cattle succumb to infection or are sent for slaughter, and whether previously infected animals can be re-infected.

The gaps in our understanding of H5N1 transmission between states, between farms, and between individual animals engender further uncertainty: the potential for H5N1 to establish in US dairy cattle long term depends on these modes of transmission and reinfection and the feasibility of disrupting them.

### Potential for spillover to swine

Swine are a natural host of influenza A viruses and lineages of H1N1, H1N2, and H3N2 subtype viruses, derived from incursions of human and avian viruses, circulate in commercial pigs (33, 34). The spread of the H5N1 virus to pigs would create the potential for co-infection and genetic exchange through reassortment, a process known to drive host switching and pandemic virus emergence (35, 36). Pigs are furthermore a demonstrated intermediary host facilitating mammalian adaptation and the transmission of novel influenza A viruses to humans (37, 38).

Swine reared in commercial agricultural, smallholder, and backyard settings are of primary relevance to influenza A virus emergence into humans, owing to the intensive interface between farmed pigs and humans. Feral swine are also susceptible to influenza A virus infection, however, and their role in the ecology of H5N1 in wildlife is incompletely understood. US commercial swine are routinely surveilled for influenza A viruses. In contrast to commercial premises, backyard settings are less systematically surveilled and may comprise several susceptible species, including dairy cattle, poultry, and pigs. Indeed, recent detection of H5N1 in pigs was associated with infection of poultry within the same backyard premises (39). Although detection of H5N1 in pigs to date is rare, concern remains. These animals have been shown experimentally to be susceptible and to support onward transmission of H5N1 viruses related to those circulating in dairy cattle (40). Epidemiological links between commercial dairy and swine premises furthermore exist, including workers, veterinarians, and shared equipment that move between sites. Despite important insights from experimentation and surveillance in swine, the potential for H5N1 to achieve sustained transmission in pigs, either prior to or after adaptation to humans, is not known. Similarly, the likelihood that reassortment of swine-lineage and H5N1 influenza viruses would give rise to novel strains of sufficient fitness to spread in pigs or spill over into humans is unclear.

### Frequency of spillover to humans

As of December 2024, 61 documented human infections had resulted from the outbreak of H5N1 in US dairy cattle (22). Most of these infections were associated with direct exposure to lactating cows or poultry that were infected by the cattle virus, although two documented cases have no known route of exposure. The proportion of human cases that are detected is, however, not clear, owing to low coverage in most states of H5N1 surveillance targeting dairy workers. A serosurvey of 115 dairy workers in Michigan and Colorado revealed evidence of infection in 7% of participants, with half reporting no memory of illness (41). These results indicate that both symptomatic and asymptomatic infections have occurred in higher numbers than documented. Widespread testing of humans with occupational exposure is needed to fully understand the frequency of spillover into humans and to understand the selective pressures placed on the virus when replication in humans does occur. Testing of their close contacts is critical to assess onward transmission from those with zoonotic infection.

### Disease in humans

Most human cases stemming from the outbreak of B3.13 genotype H5N1 in bovines have shown mild disease, often with ocular symptoms and little or no respiratory involvement. This spectrum of disease stands in sharp contrast to what has been seen with ancestral H5N1 lineages, where acute respiratory distress and death were common in human cases (42, 43). It is possible that the predominantly mild human disease documented in the USA stems from increased reporting of non-severe cases compared to prior outbreaks in Southeast Asia and North Africa. However, serological surveys revealed only low rates of positivity in persons exposed during 1997–2020, suggesting that mild infections were rare prior to the expansion of the 2.3.4.4b lineage (44, 45). The frequent use of oseltamivir to treat detected cases in the USA may also influence the observed spectrum of disease (46–48). Through the actions of cross-reactive antibodies and T cells, prior exposures to seasonal influenza viruses are thought to mitigate disease upon H5N1 infection. The extent of such protection may differ in the current outbreak relative to prior ones owing to changes in seasonal influenza viruses. Conversely, the shift from acute respiratory distress to conjunctivitis may be driven by differences in viral tropism between ancestral viruses and the B3.13 genotype circulating in dairy cattle, which derives four of its eight gene segments from North American low-pathogenicity avian influenza viruses (28). Of note, two zoonotic infections in late 2024 involving avian-derived 2.3.4.4b H5N1 viruses of the D1.1 genotype resulted in severe disease, with one of the affected individuals succumbing to infection (49, 50). These events raise concern about the consequences for human exposure of the January 2025 introduction of D1.1 virus into dairy cattle.

Mild disease in humans infected with B3.13 viruses contrasts with what has been seen in ferret and mouse models of infection and in wild and peri-domestic mammals in which infection has been detected. Ferrets infected in the lab typically show severe respiratory disease, systemic spread, neurologic signs, and death (51, 52). Wild mammals infected with 2.3.4.4b H5N1 viruses are typically detected because they succumb. Similarly, the death of infected farm cats has been observed repeatedly within the cattle outbreak (18). The drivers of these differing pathologies are unclear, but route of exposure may contribute: infection of cats and wild, carnivorous mammals is presumed to occur through feeding on infected birds. In a cynomolgus macaque model where three routes of infection were compared, however, intratracheal delivery resulted in severe disease, intranasal instillation produced more mild clinical signs, and an orogastric route did not consistently lead to infection (53). Among the signs of disease observed in both mammalian and avian species, severe neurological involvement is common (54). The potential for this aspect of disease to extend to humans is not clear.

### Extent of spillback to and circulation within wild animals

Surveillance of wildlife for influenza A viruses is sparse. Thus, although B3.13 viruses have not been detected in the wild, the possibility that these viruses from cattle have become established in wild species cannot be excluded. The genetic changes that coincided with introduction into cattle may decrease viral fitness in wild birds, which would lower the potential for maintenance of the virus in the wild, but this is unclear. Spillback into wild birds is of primary importance, since it defines the potential for further reassortment with low-pathogenicity avian influenza A viruses and for long-range dissemination of cattle-derived H5N1 viruses in migrating birds or other hosts. Spillover into wild mammalian hosts is also a concern for regional dissemination, given the broad host range of 2.3.4.4b lineage H5N1 viruses and the propensity for farms to draw in wildlife.

### Genetic diversity

As of December 2024, available sequence data, largely comprised of the >1,100 genomes generated by the National Veterinary Services Laboratories (NVSL) of the USDA, indicate that the H5N1 viruses driving the dairy cattle outbreak comprise a single genotype (B3.13). After the initial submission of this text, in January 2025, a second genotype (D1.1) was confirmed in US dairy cattle (29). Despite widespread circulation, the primary B3.13 lineage has shown relatively little change across the genome. These data are important for monitoring changes in the virus that would signal heightened risk to humans.

Even with low diversity, viral genomes carry mutational signatures that are valuable for reconstructing transmission chains. USDA APHIS Veterinary Services are conducting such analyses in real time and rapidly sharing the results with state animal health officials who bear the burden of response. The sequence data are leveraged to conduct epidemiologic tracing but cannot be made public until the investigation is considered complete by the state. Sequence data generated by NVSL are uploaded to a public database once the analyses have been shared with the state officials, but do not include information on state and date of sampling until such investigations are completed. For this reason, molecular epidemiological approaches cannot be pursued by external scientists, such that insight into paths of transmission—between cattle, between farms, between states, and between species—is lacking from the public domain.

### Evolutionary potential

**Fig 3:**
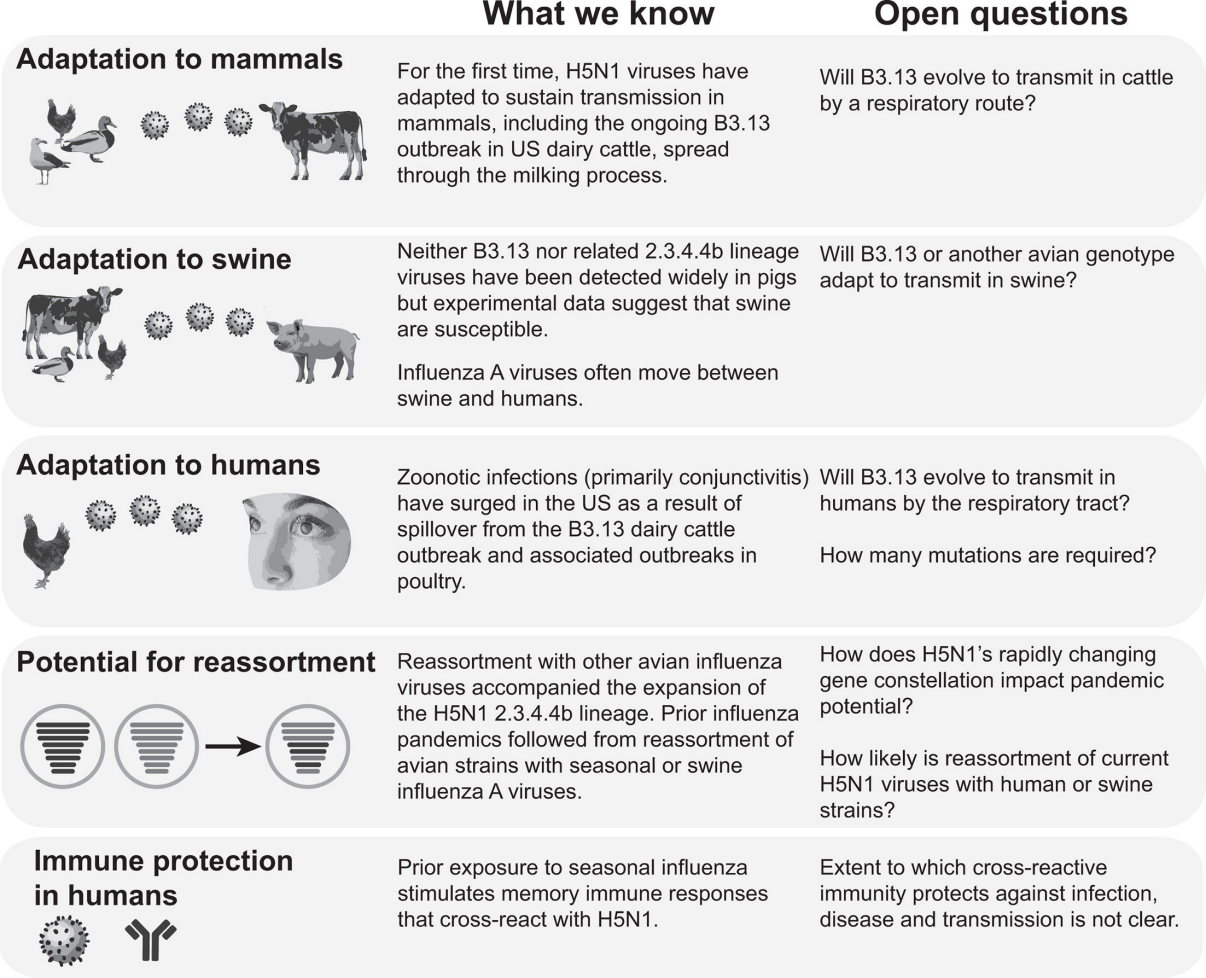
Critical open questions relate to the potential for viral evolution in different host species to increase the potential for human-to-human transmission via a respiratory route.

Influenza A viruses must adapt to human hosts to cause a pandemic. The potential for a given lineage to undergo such adaptive evolution is therefore a major factor defining pandemic risk. Several gaps in our understanding impede efforts to assess evolutionary potential (Fig. 3).

#### Adaptation to non-human mammals

The recurring spillover of 2.3.4.4b H5N1 viruses into a diverse range of mammals and, even more so, the sustained transmission of these viruses in dairy cattle (25, 28, 55), sea lions, seals (56, 57), and mink (58, 59) have been noted as dangerous opportunities for adaptation to mammals. This concern is driven by an assumption that adaptation of H5N1 viruses to any mammal increases risk to humans. The validity of this assumption is unclear. It is rooted in the close relationship between influenza A viruses circulating in swine and humans, but the relevant commonalities between these two hosts may not extend to other mammals. Indeed, beyond the glycan receptors lining the respiratory tract (60), we do not have a full understanding of which features of humans and pigs are important for the sharing of influenza A viruses between these species. Important questions for risk assessment arise: does viral replication in bovine mammary tissue and other affected mammals impose selection that will make the virus more fit in humans? Which species are of greatest concern? The potential for the dairy cattle outbreak to produce viruses with heightened transmissibility is particularly uncertain due to a lack of clarity on the modes of viral transmission among cattle. Specifically, if milking equipment mediates transmission, selection pressures are likely distinct from those active during natural transmission between mammalian hosts. Fortunately, the opportunity for reassortment in dairy cattle and most of the wild and peri-domestic mammals affected by the 2.3.4.4b panzootic is minimal, as these species often do not host sustained circulation of influenza A viruses. However, the presence of an interface between cattle and reservoir host species may vary with season and geographic location, and such interfaces would elevate concern.

#### Adaptation to humans

The impact of zoonotic infections with 2.3.4.4b H5N1 to date is mitigated by the mild disease seen in most cases. Symptoms have frequently been confined to conjunctivitis. The occurrence of these infections nonetheless remains a major concern for public health in that each is an opportunity for the virus to undergo evolution that could enable greater spread within a human host (e.g., from ocular tissues to the respiratory tract) and/or increase the likelihood of transmission to contacts. Viral replication in the eye is, however, unusual for human influenza, and how the selective environment of the eye compares to that of the respiratory tract is uncertain. This is important since viral adaptation to the human eye may or may not increase the risk of a respiratory pandemic. The unusual tropism of 2.3.4.4b H5N1 viruses in humans may furthermore limit the opportunity for reassortment with seasonal strains, as these viruses are not known to replicate in the tissues of the eye (61).

#### Reassortment

Unlike mutation, reassortment with a divergent virus confers major genotypic change in a single replicative cycle. This mechanism of genetic diversification can thereby accelerate adaptation. Although influenza A viruses in wild birds reassort frequently (62), the patterns of reassortment that accompanied the geographic expansion of the 2.3.4.4b lineage are notable (63). The H5 hemagglutinin has been paired with multiple different genetic backgrounds derived from low pathogenicity avian influenza viruses. Whether this reassortment drove expansion by increasing fitness in wild avian species that occupy differing geographic ranges is not clear. Whether the reassortment that yielded the B3.13 genotype circulating in cattle was important to the introduction of the virus into this species is also not known. Importantly, how the reassortant genotypes circulating in different species and regions differ in their potential for adaptation to humans and pandemic spread is not understood.

Past influenza pandemics suggest that reassortment of H5N1 influenza viruses with human- or swine-adapted strains is of great concern. The likelihood of reassortment with seasonal strains increases with their prevalence, and thus, the risk of H5N1 viruses reassorting within human hosts will peak in the North American winter. Spillover from cattle or wild animals to swine at any time of year would engender significant risk of reassortment. Importantly, however, reassortment is unlikely to immediately produce a pandemic virus.

The abrupt combination of genes from divergent influenza A viruses typically results in mismatches among viral components, such that reassortant viruses suffer fitness defects (64, 65). This negative epistasis can be overcome by post-reassortment adaptive change (66), but a low-fitness reassortant may go extinct before this process plays out. These considerations infuse uncertainty into discussions of the potential for a pandemic strain to arise through reassortment. What range of fitness effects are associated with the reassortment of H5N1 viruses with strains circulating in humans or pigs? How many mutations would be needed to overcome any negative fitness effects? Owing to the complexity of interactions among viral gene segments and the sparsity of prior work examining these questions across the diversity of influenza, the extent to which the fitness effects of reassortment can be anticipated is extremely limited.

#### Barriers to host switching

Since viruses rely on cellular machinery to produce progeny, replication in a new host species presents a multitude of barriers. For influenza A viruses, some of these barriers are well understood, such as host-specific glycans that are needed for viral entry (67). Our knowledge is limited, however, in two ways. First, there are barriers to host switching that have not been defined mechanistically. Second, the genetic/phenotypic changes needed to overcome even well-defined host restrictions are often unclear or narrowly defined. These fundamental knowledge gaps significantly hinder efforts to anticipate and monitor for evolutionary change that would increase the fitness of H5N1 influenza virus in humans.

### Extent of protection derived from prior exposure to seasonal influenza

To spread widely in humans through respiratory transmission, an influenza A virus must be sufficiently novel antigenically to invade the ecological niche occupied by seasonal influenza viruses. H5N1 subtype avian influenza viruses are distinct from seasonal H3N2 and H1N1 subtypes but carry some common antibody and T cell epitopes that may limit their pandemic potential (68–70). These shared epitopes almost certainly mitigate disease in infected persons who have pre-existing immunity to seasonal influenza.

Reactivity of antibodies elicited by seasonal influenza infection or vaccination to H5N1 viruses has been assessed for a range of age groups, representing differing exposure histories (47, 71). The levels of protection against infection and disease conferred by such cross-reactive antibodies are, however, not clear. Experiments in ferrets give some insight: prior infection with seasonal virus strongly suppresses H5N1 viral replication and gives full protection against severe disease, with seasonal H1N1 being more protective than H3N2 (46, 48). Important gaps not addressed by experimental studies to date are the duration of protection conferred by seasonal influenza infection and the extent of protection achieved through seasonal influenza vaccination.

## WHAT LIMITATIONS IMPEDE ASSESSMENT OF THE PANDEMIC RISK POSED BY THE BOVINE H5N1 OUTBREAK?

**Fig 4:**
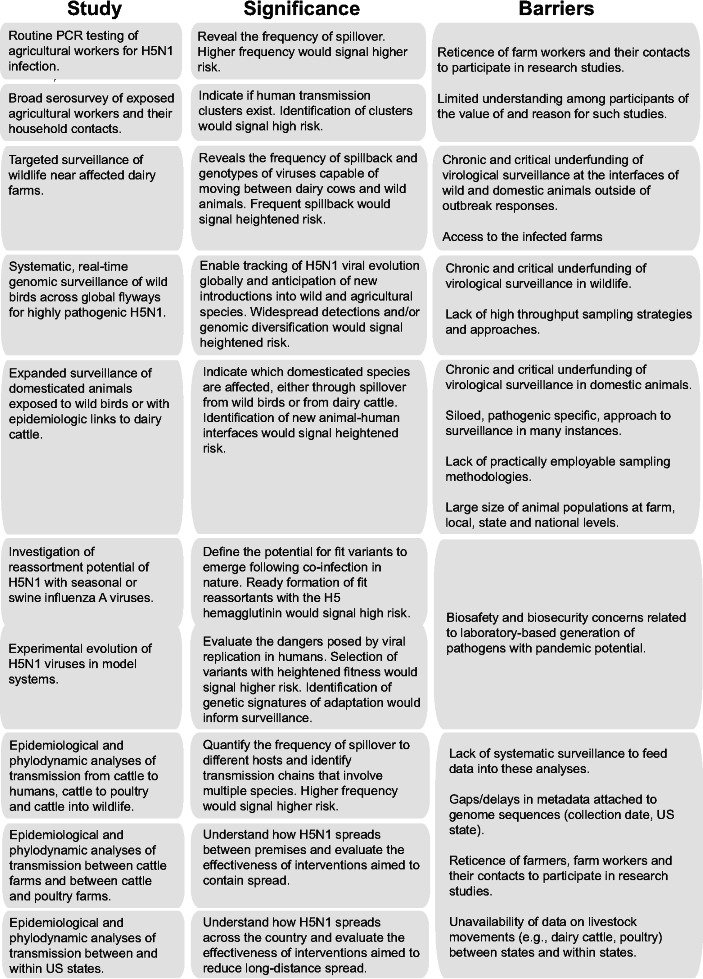
Limitations on key research that would advance assessment of H5N1 pandemic risk.

Barriers to research that would improve our ability to anticipate or prevent an influenza pandemic derive from multiple sources (Fig. 4).

### Constraints on surveillance

Virological and serological surveillance efforts are reliant on partnerships with stakeholders outside of the influenza research community. Field scientists work with government, landowners, farmers, veterinarians, medical doctors, researchers in other fields, etc. to secure access to populations of wildlife, domestic animals, and humans with exposure to these animals. These wildlife and domestic animal interfaces are sectors that are chronically and critically underfunded. The willingness of these partners to grant access often hinges on sensitive issues related to the maintenance of public relations, livelihoods, animal health and welfare, farm operations, and business opportunities. These issues have figured prominently in the bovine H5N1 outbreak, where animal health and welfare and continuity of milk production are counterbalanced with detecting or limiting viral spread. The sensitivity of testing dairy workers is especially high due to the large proportion of these at-risk individuals who are immigrants and may be part of communities with undocumented individuals. Such persons are often less likely to seek medical care or trust public health officials.

Even on farms that are open to sampling and testing, surveillance at a scale that is adequate to support epidemiological investigation is difficult to achieve. When considering the large number of animals involved in many commercial dairy operations, current sample collection strategies are impractical to apply at the level of individual cattle. Research into novel sampling strategies is needed to overcome this obstacle.

Surveillance of wastewater for viral RNA offers a relatively unintrusive means of collecting data on viral prevalence in an area. At present, however, this approach cannot decipher the sources of viral RNA detected. Thus, H5N1 RNA detection in wastewater could be derived from infected humans, various species of wild or domesticated animals, or discarded pasteurized milk rather than an actively infected dairy.

### Limitations on availability of containment facilities for research in natural hosts

There are only a handful of laboratories in the United States capable of handling large animals at biosafety level 3 containment, which severely limits scientific advancement. Even within the facilities that can house cattle, providing husbandry to lactating cows weighing nearly 650 kg and consuming >45 kg of feed per day is logistically difficult. Demands on resources, personnel, space, and specialized equipment are all high. None of these facilities were built with milking systems in place. Studies using lactating cows are therefore restricted to small numbers of animals, which both limits statistical power and stands in contrast to modern dairy farm settings, which often house thousands of animals.

### Limitations on molecular analysis of transmission routes and evolutionary dynamics

The application of molecular epidemiology and phylodynamics to the dairy cattle outbreak has been slowed by constraints on rapid public sharing of data, including viral sequences and associated metadata. Analysis of these data can elucidate routes of H5N1 viral dissemination among cattle and other affected species and reveal patterns of evolution indicative of increased zoonotic risk. The sequences of H5N1 positive samples are being determined by US Department of Agriculture scientists as part of their response to the outbreak. However, data release is governed by individual states, creating delays and state-by-state variation in data availability that hinder comparison across states and the identification of changes in outbreak dynamics.

Further constraints arise with GISAID, a database that houses the bulk of consensus viral sequences relevant to the bovine H5N1 outbreak. The management of user access to this database is inefficient and lacks transparency, such that scientists seeking to contribute to outbreak response research may not be able to download data shared by their colleagues with GISAID. One consequence is that relevant data are now strewn across several different databases, which operate independently, contain different data types in different formats, and sometimes contain duplicate entries that lack the information needed to identify them.

### Challenges to assessing risk based on viral sequences

A handful of viral phenotypes and their associated genotypes that are important for adaptation to humans have been described. This knowledge is the cornerstone of efforts to distill risk from surveillance and viral genomic data, and few of these known risk factors have been identified in bovine H5N1 viruses. There are blind spots in this approach, however, which come in two forms. The first constitutes gaps in our understanding of the viral traits necessary and sufficient to drive pandemic spread. Sustained research focused on discovery is needed to resolve these blind spots. The second is a disconnect between determination of viral genotypes and understanding of viral phenotypes. While viral genome sequencing can be carried out with high throughput, translating the resultant information to estimate risks to humans is reliant on phenotypic characterization of viral isolates, often involving slow and complex work in the laboratory. To advance risk assessment during a rapidly evolving outbreak, assays that yield phenotypic data relevant to risk and that can be carried out in higher throughput are needed.

### Challenges to identifying viral phenotypes associated with pandemic potential

Research focused on evaluating the risk that an outbreak will occur is necessarily reliant on model systems, as working in natural systems would require that the outbreak unfold. Every model, whether experimental or computational, has limitations. Models can nonetheless be extremely useful if appropriately designed for the research question and interpreted with consideration of caveats. In the context of the bovine H5N1 outbreak, the following limitations of research models are prominent.

Serological analyses indicate that humans carry antibodies that recognize the dairy cattle H5N1 viruses. A major limitation to the application of this information for epidemic modeling, however, is translating serological data to estimates of protection—from infection, severe disease, and onward transmission.

Ferrets have served as a valuable model for evaluating influenza virus pathogenicity and transmission, often with clear relevance for human infection (72, 73). However, in contrast to the mild disease seen in infected humans, challenge of ferrets with B3.13 genotype H5N1 viruses derived from the dairy cattle outbreak has typically resulted in rapid progression to death (51, 52). Inoculation of ferrets at the eyes, to model ocular exposure of dairy workers, yields little replication in eye tissues. Rather, this approach quickly results in viral replication within the respiratory tract and a course of disease like that seen with intranasal challenge (74). Thus, further refinements—and potentially different model species—are needed to address questions relevant to human disease. Efforts to use ferrets or other animal models to assess transmission potential are further frustrated by uncertainty related to how the virus transmits in the field—between cows, from cows to humans, and potentially from human-to-human. The modes of viral shedding and exposure that are relevant to both mammary tropism in cows and ocular tropism in humans are unclear, impeding model design.

### Barriers to assessing viral evolutionary potential

Research limited to the characterization of extant influenza A viruses fails to grapple with the fact that pandemics are driven by viral evolution. Examination of viruses that could readily evolve from circulating strains—through acquisition of a small number of mutations or through reassortment—is essential to understanding pandemic risk. However, constraints on studying genetically altered viruses limit such research. These constraints come in two forms: the conservative implementation of regulatory frameworks for oversight of pathogen research (75, 76) and a public discourse that is hostile to virology research in the wake of the COVID-19 pandemic (77). These factors prevent or deter scientists from investigating the potential for viral acquisition of phenotypes associated with risk to humans.

## CONCLUSION

Meaningful assessment and effective mitigation of pandemic risk depend on a thorough understanding of the drivers of viral emergence and spread across animal and human populations. Both rapid outbreak-response and sustained research are needed to furnish the information required. At present, a deepening of the research response to the ongoing H5N1 panzootic will help to define the scope of the outbreak and the nature of human exposure. The knowledge gained will enable the design of biosecurity measures to diminish H5N1 circulation in animals, thereby reducing human exposure. Now and over the long term, research is needed to better define the factors that allow influenza pandemics to occur. While certain features are well-defined, uncertainty remains as to whether a given zoonotic threat can trigger a pandemic. Such research will enable more accurate assessment of risk, in turn allowing targeted preparation of antivirals, vaccines, and other interventions that can reduce the impact of an outbreak.
